# Feasibility and Safety of Implementing Volumetric Arc Therapy (VMAT) for Pediatric Craniospinal Irradiation in a Low-Middle-Income Region: The Nigerian Experience

**DOI:** 10.1016/j.adro.2023.101325

**Published:** 2023-07-28

**Authors:** Adedayo Joseph, Adeseye Akinsete, Samuel Adeneye, Onyinye Balogun, Opeyemi Awofeso, Aishat Oladipo, Azeezat Ajose, Ibrahim Elhamamsi, Kenneth Merrell, Wilfred Ngwa, David Puthoff, Abdul R. Shour, Adedayo Onitilo

**Affiliations:** a NSIA-LUTH Cancer Center, Lagos University Teaching Hospital, Lagos, Nigeria; b Department of Pediatrics, College of Medicine, University of Lagos, Lagos, Nigeria; c Department of Radiation Biology, Radiotherapy and Radiodiagnosis, College of Medicine, University of Lagos, Lagos, Nigeria; d Department of Radiation Oncology, Weill Cornell Medicine, New York, New York; e Psychosocial Oncology & Palliative Care, Dana Farber Cancer Institute, Boston, Massachusetts; f Department of Radiation Oncology, Mayo Clinic, Rochester, Minnesota; g Sidney Kimmel Comprehensive Cancer Center, School of Medicine, Johns Hopkins University, Baltimore, Maryland; h Office of Research and Sponsored Programs, Marshfield Clinic Research Institute, Marshfield Clinic Health System, Marshfield, Wisconsin; i Cancer Care and Research Center, Marshfield Clinic Research Institute, Marshfield Clinic Health System, Marshfield, Wisconsin; j Department of Oncology, Cancer Care and Research Center, Marshfield Clinic Health System, Marshfield, Wisconsin

## Abstract

**Purpose:**

Volumetric modulated arc therapy (VMAT) is a relatively new treatment technique in sub-Saharan Africa. Although craniospinal irradiation (CSI) in the pediatric population has been practiced in Nigeria for many years, the use of VMAT to deliver this treatment is previously undocumented. We reviewed the first set of patients to undergo CSI at a cancer center in Nigeria, detailing the treatment technique, the progress experienced, dose statistics achieved, treatment toxicities, and cancer outcomes to date.

**Methods and Materials:**

This was a prospective case series of 5 children with histologically diagnosed cancers requiring CSI whose parents consented to the study. They were recruited at evaluation and followed through the process of their therapy. Toxicity was monitored at weekly review appointments using the Common Terminology Criteria for Adverse Events version 5.0. Follow-up of the children will continue in the long-term effects clinic.

**Results:**

Five patients with a median age of 6 were recruited. Diagnoses were intracranial germ cell tumor (n = 2), medulloblastoma (n = 1), pineoblastoma (n = 1), and ependymoma (n = 1). For all patients, a dose of 36.0 Gy in 1.8 Gy daily fractions was prescribed to the entire neuraxis. A subsequent boost of 18 Gy (n = 4) to 19.8 Gy (n = 1) in 10 daily fractions to the primary tumor bed (n = 2) and posterior fossa (n = 2) was delivered. Four patients had chemotherapy before, during, or after radiation therapy. No patient experienced grade 3 or greater toxicity.

**Conclusions:**

Our results indicate great progress has been made in the delivery of CSI in Nigeria, demonstrating tolerable acute side effects using VMAT. This series suggests the feasibility of implementing VMAT technology in low- or middle-income countries. Additional follow-up will be needed to determine whether survival rates and chronic toxicity rates are similar to those reported in the literature.

## Introduction

### Background

Primary central nervous system (CNS) tumors are the second most common cancers in the 0- to 19-year age range, accounting for up to 25% of all malignancies in children and adolescents.^1^ In Nigeria, primary CNS tumors are the fourth most common malignancies in the pediatric (0-14 years) population. Of these, astrocytic tumors and medulloblastomas (MBs) are the most common.^2^ Globally, MB is the most common malignant CNS tumor in children, constituting 20% of primary brain tumors and approximately 40% of all tumors of the posterior fossa, whereas astrocytoma and ependymoma make up the second and third most common.^3^^,^^4^ Globally, pediatric patients with CNS tumors have seen significant improvement in survival and side effect outcomes since the early 1960s because of advances in treatment techniques and technology.

CNS tumors that seed through the cerebrospinal fluid (CSF) require treatment of the entire neuraxis in a radiation therapy (RT) technique called “craniospinal irradiation” (CSI).^5^ Most commonly employed for the multimodal treatment of MB, CSI is also indicated in the definitive treatment of many cranial tumors. These tumors include CNS leukemia, germ cell tumors, ependymomas with evidence of CNS involvement, and multicentric CNS lymphoma.^6^^,^^7^ Other tumors treated with CSI are intracranial germ cell tumors such as germinomas, ependymoma, atypical teratoid rhabdoid tumors, pineoblastoma, central neurocytoma, glioneuronal tumor, gliomas, choroid plexus carcinoma, and leukemia.^6^^,^^8^^,^^9^

Significant improvements in understanding tumor biology, treatment regimens, and advancement in RT techniques have led to increased survival rates and reduced toxicity in the management of previously fatal conditions like MB.^10^ Side effects of RT are volume-, dose-, and age-dependent and are a result of unrepaired sublethal damage to tissue exposed to radiation. Common acute side effects include nausea, vomiting, esophagitis, diarrhea, myelosuppression, fatigue, and weight loss. A large volume of bone marrow and circulating blood are within the CSI field, which may result in significant acute hematological toxicity.^6^^,^^11^ The evolution of RT techniques has improved the accuracy of treatment delivery and reduced acute and long-term toxicity and sequelae. In high-income countries, intensity modulated RT (IMRT), volumetric modulated arc therapy (VMAT), and proton beam RT are commonly used as the standard of care.^5^ The VMAT technique, in particular, has the advantage of delivering radiation at optimal target coverage with significantly reduced treatment times and sparing of normal tissues/organs at risk (OAR), and it is becoming more available in low- and middle-income countries (LMIC) nations like Nigeria (E. Uche, et al, unpublished data, 2019).^6^^,^^12^, ^13^, ^14^

Nigeria and other sub-Saharan countries continue to record disproportionately low survival rates from pediatric CNS tumors. Uche et al^15^ in a 2020 review of cases in the southeastern region in Nigeria showed that pediatric brain tumor cases treated with RT had poor overall survival rates, with 40% and 0% at 1- and 5-year survival, respectively. Similarly, in a single institutional review of a cancer institute in Sudan, the 2- and 5-year survival rates were 33% and 13%, respectively.^16^ RT in Nigeria has evolved from telecobalt therapy to modern linear accelerators (LINACs) at multiple centers with capability for IMRT and VMAT.^17^ This modern conformal technique with better dose delivery profiles and normal tissue protection may improve cancer outcomes for pediatric patients in Nigeria. Here, we report the first documented experience of delivering VMAT in the country with the first set of pediatric patients to undergo CSI via VMAT at a cancer center in Lagos, Nigeria, detailing the treatment technique, progress experienced, doses achieved, treatment toxicities recorded, and current status of the treated patients.

## Methods and Materials

### Patient selection

After institutional ethical approval, the first 5 patients under 18 years with histologically diagnosed cancers to receive CSI delivered via VMAT were recruited with parental and patient consent between September and December 2019 and followed through the process of their therapy. All 5 had received some surgical intervention, including ventriculoperitoneal shunt insertion, biopsy, or surgical resection before referral to our center for RT. Four patients received chemotherapy at different times in their treatment: neoadjuvant (n = 3) and concurrent with RT, followed by adjuvant (n = 1).

Demographic, diagnostic, and clinical information were extracted from clinic case files and records. Treatment plan and dose distribution data were extracted from the treatment planning system (TPS) software. Acute side effects were monitored at weekly RT review visits and recorded in the case files from which they were extracted.

### Radiation treatment

All patients were simulated in the supine position using a computed tomography simulator. The patient's head was supported by a suitable headrest with maximum neck extension to reduce exit dose through the oral cavity and immobilized with a thermoplastic mask. The body was immobilized with a vacuum bag, knee wedge, and ankle support. Play and exposure therapy techniques were used before and during simulation to put patients at ease and ensure they knew what to expect at every step of the process.

### Planning

Target volumes and OARs were identified and contoured following available atlases and guidelines such as the European Society for Paediatric Oncology (SIOPE) brain tumor group consensus guidelines.^18^, ^19^, ^20^ The clinical target volume (CTV) of the brain and spine were contoured as separate structures using both bone and soft tissue windows in all views (axial, sagittal, coronal) to ensure adequate coverage. The CTV brain was contoured to cover the entire cranial compartment around the brain, taking care to cover the CSF spaces around the cribriform plate and between the orbits. The CTV spine volume was the spinal canal without the vertebral bodies, contoured on bone windows to ensure adequate coverage of the neuro foraminal exits. A thecal sac structure was created, and the CTV spine contoured to 2-cm below the edge of the thecal sac as identified on magnetic resonance imaging. The 2-cm extension was selected to reduce the risk of undercontouring the spinal volume in this pilot set of patients. Contouring was done by the radiation oncologist following contouring guidelines such as the SIOPE brain tumor group consensus guidelines.^18^ Peer review of the contouring and planning was done to ensure optimal contouring and reduce the risk of errors by a visiting expatriate radiation oncology support team from Egypt. Each plan was peer-reviewed, and contours were reviewed to ensure errors such as inadequate coverage of the neuroforamina, minimal areas of inclusion of the vertebral body in the CTV spine, and undercoverage of the CTV brain volume around the crista galli were avoided or corrected. OARs, including the bilateral lenses, retina, eyes, optic nerves, cochleae, lungs, kidneys, testes, parotid glands, optic chiasm, thyroid, esophagus, heart, liver, bowel, and bladder were contoured for RT planning.^18^^,^^20^

VMAT RT was planned in 2 phases. Phase I treated the entire neuroaxis planned target volume (PTV), which included the CTV brain with a uniform 0.7-cm PTV margin, and the CTV spine with a PTV margin of 1.0 cm to a dose of 36 Gy (n = 5) at 1.8 Gy per fraction. The larger PTV spine margin was chosen in consistency with data showing higher variability with spine setup and immobilization, as well as to ensure adequate and uniform coverage of the entire vertebral bodies to reduce long-term scoliosis/kyphosis.

The second phase of treatment consisted of a boost to the posterior fossa (n = 2) or tumor (n = 3). Pre- and postoperative magnetic resonance images were imported and registered to simulation images to aid in delineation. The boost delivered an additional 19.8 Gy (n = 1) and 18 Gy (n = 4) in 1.8 Gy per fraction. Patients were treated with 6 and 15 MV photon beams from a 120 multileaf collimator LINAC.

**Figure 1:**
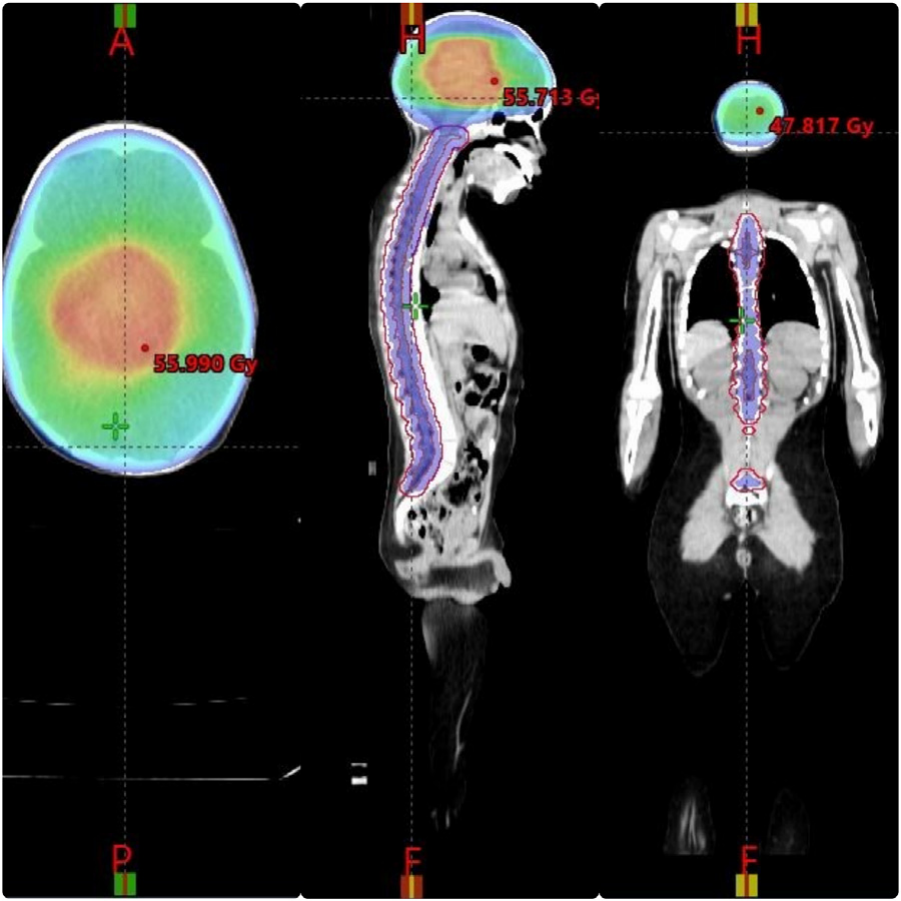
Dose distributions in sagittal and axial views. Dose color wash images in the sagittal and axial views, with relative dose color wash from 50% to 95% of the prescription dose.

**Figure 2:**
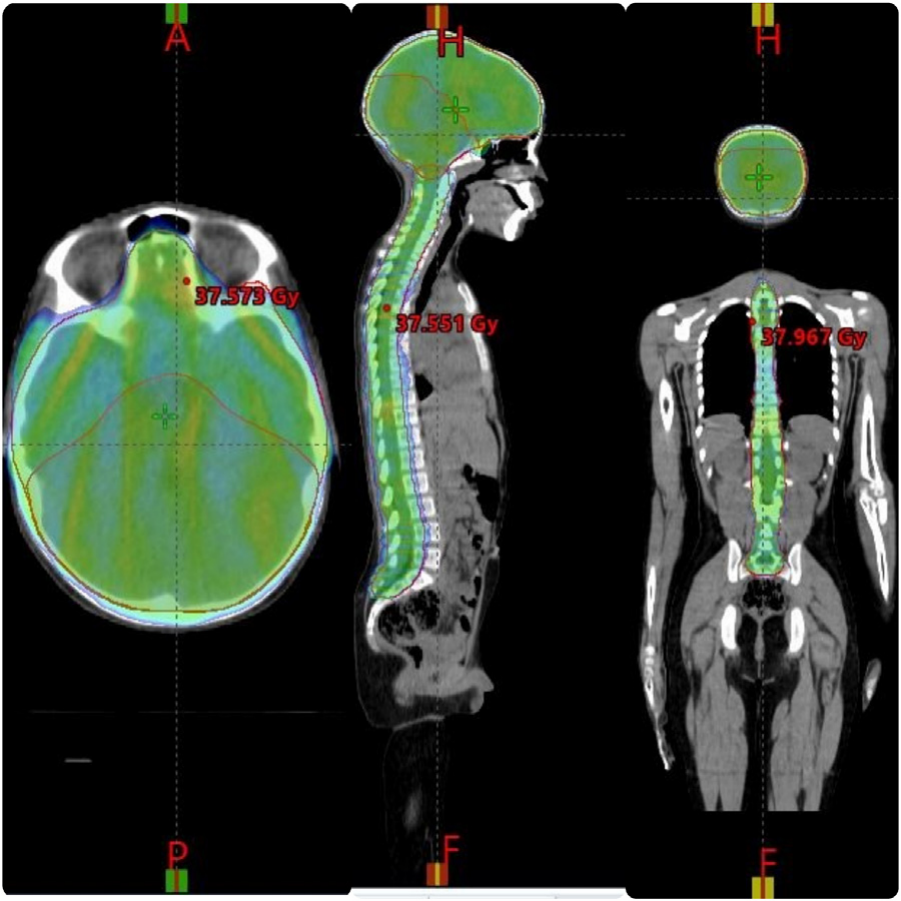
Dose distributions in sagittal and axial views. Dose color wash images in the sagittal and axial views, with relative dose color wash from 50% to 95% of the prescription dose.

**Figure 3:**
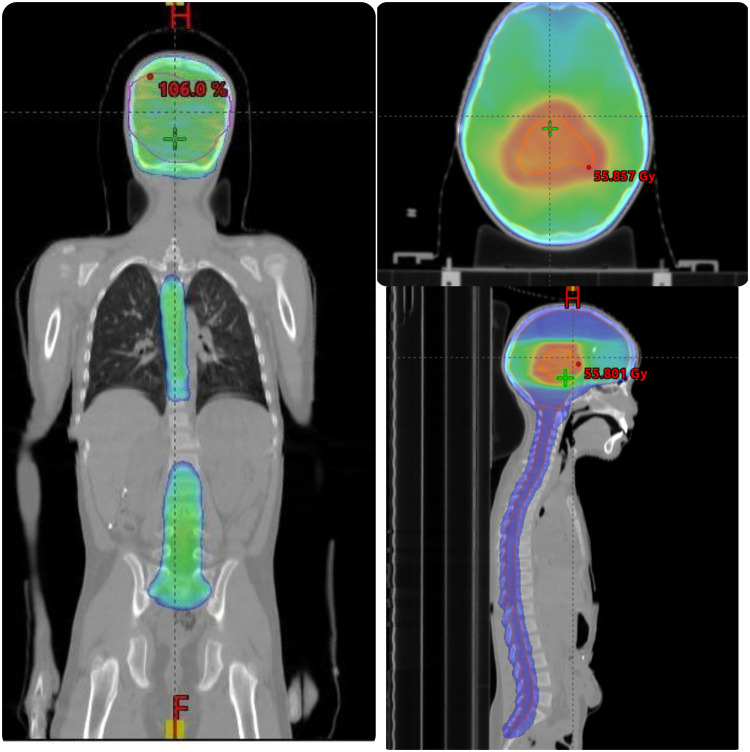
Dose distributions in sagittal and axial views. Dose color wash images in the sagittal and axial views, with relative dose color wash from 50% to 95% of the prescription dose.

Patients were treated with 6-MV photon beams from a Varian LINAC “VitalBeam” equipped with 120-leaf multileaf collimator. The whole neuraxis was planned using VMAT. On the arc geometry tool, 3 isocenter 3 full rotation with a target margin of 0.5 cm was selected, as shown in Figs. 1, 2, and 3. All patients had isocenters placed at the middle of the brain, at the level of T5 with 22 cm of separation and the third isocenter at the level of L3 with 20 cm of separation from T5 level. The first isocenter consisted of 3 arc and 10° collimation with a starting angle of 179° and an ending angle of 181°. Second isocenter consisted of 2 arc and 10° collimation with a starting angle of 179° and an ending angle of 181°. Third isocenter consisted of 2 arc and 10° collimation with a starting angle of 179° and an ending angle of 181°. These plans were optimized according to institutional practice. To increase dose conformity and control the dose gradient outside of the PTV, a ring control structure (0.3-cm inner wall margin and 3-cm outer wall margin from the PTV total) was built. The ring control structure was assigned upper objectives with a constraint of 0% volume of the ring not receiving 95% of the prescribed dose with a priority of 180. All arcs were optimized simultaneously, and the plan was optimized by imposing dose-volume limits on the PTV, OAR, and control structure. In addition, the normal tissue objective tool in Eclipse was used to minimize the dose outside the target normal tissue objective “NTO” even further. The plan was optimized using the progressive resolution optimizer techniques provided in Eclipse (version 15.6.05), and the dosage was calculated using the anisotropic analytical algorithm with a grid size of 2.5 mm (V 15.6.05). After evaluation, the 2 isocenters were separated into 2 plans.

Target coverage was expressed as the PTV receiving at least 95% of the prescribed dose (D95%) and the volume receiving more than 107% of the prescribed dose (V107%). Dose color wash images in the sagittal and axial views are presented in Figs. 1, 2, and 3, with relative dose color wash from 50% to 95% of the prescription dose. The dose-volume histogram was reviewed for the PTV coverage to 97% of the prescribed dose and average dose to the OAR such as lenses (maximum dose 5-11 Gy), heart (mean dose < 15 Gy), esophagus (mean dose < 15 Gy), kidneys (mean dose < 10 Gy), parotids (mean dose < 12 Gy), thyroid (V26 Gy < 10%), and liver (mean dose < 8 Gy). A verification plan was created by the TPS using the electronic portal imaging device (EPID). For plan quality comparison, the dose homogeneity index (HI) and dose to OAR using the parameters obtained from the dose-volume histogram are shown in Tables 1 and 2. HI was obtained based on the definition proposed by International Commission on Radiation Units and Measurements Report 83 (ICRU-83) and also used by Adeneye et al^21^^,^^22^ as presented:

HI=D2%−D98%/D95%

**Table 1:** Mean doses of PTV and all OARs

	Case 1	Case 2	Case 3	Case 4	Case 5
PTV brain					
D2%	37.67	37.504	38.034	41.231	36.042
D98%	35.06	34.821	32.106	37.928	34.652
D95%	35.56	35.538	34.612	38.883	36.236
HI	0.0733	0.0754	0.1712	0.0849	0.03835
PTV spine					
D2%	37.464	37.154	37.237	41.373	36.63
D98%	34.475	33.581	34.39	37.901	33.782
D95%	35.128	34.631	34.994	38.383	35.365
HI	0.0850	0.1032	0.0814	0.0905	0.0805
PTV boost					
D2%	56.613	55.61	55.641	59.945	56
D98%	53.803	53.992	54.283	55.227	54.952
D95%	54.167	54.118	54.422	56.64	54.363
HI	0.05187	0.0299	0.0249	0.0832	0.0193

*Abbreviations:* HI = homogeneity index; OARs = organs at risk; PTV = planning target volume.

**Table 2:** Organs at risk

		Case 1	Case 2	Case 3	Case 4	Case 5
Bladder	D_max_ Gy	0.617	1.506	7.076	17.644	6.32
Bowel	V195 cm^3^ (Gy)	13.309	10.766	9	14.171	13
Cochlea (L)	D_mean_ Gy	52.871	38.171	37.689	59.437	42
Cochlea (R)	D_mean_ Gy	52.768	38.223	37.078	59.477	40.258
Retina (L)	D_max_ Gy	24.378	18.795	35.309	39.116	33.78
Retina (R)	D_max_ Gy	22.607	15.629	30.14	39.709	32.58
Parotid (L)	D_mean_ Gy	10.731	5.486	7.397	18.682	11.32
Parotid	D_mean_ Gy	11.578	5.578	7.657	18.117	12.88
Optic nerve (L)	D_max_ Gy	43.387	31.291	37.5	35.424	33.87
Optic nerve (R)	D_max_ Gy	43.459	29.344	41.064	34.199	35.654
Kidney (L)	D_mean_ Gy	10.56	8.186	8.703	18.09	15.25
Kidney (R)	D_mean_ Gy	8.898	7.543	8.846	16.736	9.23
Liver	D_mean_ Gy	8.386	6.236	6.208	10.335	9.235
Optic chiasm	D_max_ Gy	55	55	55	58.266	57
Lung (L)	D_mean_ Gy	8.79	7.119	9.103	14.815	7.23
Lung_R	D_mean_ Gy	8.827	5.56	9.397	14.235	8.5
Heart	D_mean_ Gy	8.19	11.398	5.133	12.72	10.25
Lens_L	D_max_ Gy	10.351	6.382	7.171	8.697	9.36
Lens_R	D_max_ Gy	10.206	6.084	7.292	8.416	6.2

*Abbreviations:* D_max_ = maximum dose; D_mean_ = mean dose.

D2% and D98% represent the minimum dose received by 2% and 98% of the target volume, indicating the maximal and minimal doses to the target, respectively, and D95% represents the dose received by 95% of the target. The closer the value of HI is to 0, the more homogenous the plan.

In general, the center sets a hard limit of 10 working days from presimulation counseling to start of therapy. For the patients in the series, the time from prescription of RT to start of treatment ranged between 8 to 11 working days. Contouring started immediately after simulation for pediatric patients and was peer reviewed within 3 working days. Planning followed a similar timeline with plan evaluation and any plan corrections aimed to be completed by working day 9 postsimulation.

### Quality assurance

The clinically acceptablethree isocentre (3-ISO) VMAT plan for each patient was subjected to patient-specific quality assurance processes to confirm beams were deliverable. All measurements were performed using an aS1200 Portal Vision EPID panel (Varian Medical Systems) and EPIDQA software v 15.6.05 (Epidos, Bratislava, Slovakia). The arrays have an active area of 40 × 40 cm with a pixel number of 1190 × 1190 and a pixel resolution of 0.34 mm at isocenter. In this study, all measured fluence maps were added together. For each radiation fraction, a summary fluence map was generated. The composite image function was used in the portal dosimetry module of the ARIA v 15.6 software (Varian Medical Systems, Palo Alto, CA). The summary maps from all other treatment fractions were compared with the corresponding summary fluence maps obtained for a single fraction. All pairs of fluence maps were used for the analysis. The similarity in fluence map pairs was assessed based on the gamma coefficient calculated in the analyzed field.

Acceptable dose difference values of 3% and dose shift value by 3 mm were assumed. Pairs of fluence maps agreed if this criteria (γ[3%,3 mm] < 1) was met in 95% of analyzed field points. The adopted gamma criteria was 3%, 3 mm, 95%. The portal dosimetry module was also used to compare fluence maps and gamma calculations. A situation in which all pairs of fluence maps met gamma criteria without exception was considered a fully repeatable irradiation of the patient. Fluence map measurements and analysis were carried out for all 5 cases in this study.

### Treatment delivery

Each patient was simulated supine on a whole-body board (Radon Medical Equipment, Yenimahalle/ANKARA) with immobilization. The simulation was carried out using GE computed tomography (Optima 580; GE Healthcare, Waukesha, WI) of 16 slices and 2.5-mm thickness. The Eclipse TPS (version 15.6.05) was used for contouring and treatment planning, and the anisotropic analytical algorithm was used for dose calculation. The patients received RT with a clinical LINAC, VitalBeam model (Varian Medical Systems), in our department. Daily treatment delivery was by a therapy team trained in pediatric behavioral and play therapy, using videos and age-appropriate education techniques to reassure patients. Sedation was not used for any of these patients using these techniques.

### Sedation

As a cancer center in a resource-limited setting, there is no dedicated pediatric anesthesia team, and limited anesthesiologists are available. Young patients needing sedation must wait sometimes several hours, often fasted and hungry, for an anaesthesiologist to become available. This shortage in some instances necessitates cancellation of a daily treatment, leading to missed fractions and prolongation of overall treatment duration. Furthermore, sedation at our center leads to an increase in the cost of treatment by an average of 1000 dollars (United States $1000). In the context of a minimum wage of $65 and treatment costs mostly paid out-of-pocket, the need for sedation can be a significant barrier to treatment.^23^^,^^24^ As such, the motivation to avoid sedation is substantial.

Our pediatric radiation team uses a protocol executed by the combination of radiation oncologist and therapist, as there are no specialist pediatric behavioral or play therapists accessible to the team. “Practice” sessions — a minimum of 1 and maximum of 3 — are employed before the simulation appointment to try to put the patient at ease. The practice (and subsequent treatment) sessions are accompanied by a rewards system with the children receiving candy, ice cream, and toys such as action figures or dolls after each session. Treatment videos available for free on YouTube are watched with the caregivers and the patient in preparation for the sessions. The first session is done with the primary caregiver (usually a parent) in the room, who is then withdrawn in subsequent sessions. The process invariably begins with significant anxiety and resistance from the patient. For most children 5 years of age and older, the team can establish some trust and comfort by the simulation session. In children who are not reassured by session 3, the anesthesia team is invited to be present, as this would then become the simulation session if sedation is necessary.

### Chemotherapy

Four patients had chemotherapy. Three patients had neoadjuvant chemotherapy before presentation for RT at our center, and 1 had concurrent weekly vincristine 1.5 mg/m^2^ (maximum of 2 mg per dose) while on RT followed by adjuvant chemotherapy at the pediatric oncology unit. Chemotherapy was administered by the pediatric oncology unit for all patients except case 2, who received concurrent vincristine while on RT at our center.

The most common chemotherapy regimen consisted of alternating cycles of carboplatin 600 mg/m^2^ on day 1/etoposide 100 mg/m^2^ on days 1 through 3 (cycles 1 and 3) and ifosfamide 1000 mg/m^2^ on days 1 to 5/etoposide 100 mg/m^2^ (cycles 2 and 4) in 3 patients (patients 1, 3, 4). Patient 2 (MB) had concurrent chemotherapy with weekly vincristine at a dose of 1.5 mg/m^2^ to a maximum of 2 mg per dose during RT at our center followed by post-RT adjuvant chemotherapy with 6 cycles of vincristine 1.5 mg/m^2^ (maximum of 2 mg per dose), cisplatin 75 mg/m^2^ with mannitol 7.5 G/L, and cyclophosphamide 1000 mg/m^2^ with mesna at 360 mg/m^2^ at the pediatric oncology unit of the hospital.

### Follow-up

Patients were evaluated by radiation oncologists at weekly follow-up visits. These evaluations included clinical history, physical examinations, and laboratory investigations. Acute toxicity was evaluated using the Common Terminology Criteria for Adverse Events (CTCAE) version 5.0. Emotional and mental states were assessed at weekly visits during treatment and every 3 months after completion of treatment. The median follow-up for all patients was 35 months (range, 32-40 months). Patient 5 was lost to follow-up and was not included in this count. Because of distance and travel constraints for patients coming from out of state, follow-up is predominantly via phone calls to caregivers.

### Statistical analyses

Data were collected in an Excel file designed for this study and imported into IBM SPSS (version 22.0 for Windows; Armonk, NY) for statistical analysis. Descriptive analysis was done using means and standard deviations for continuous data and frequencies and percentages for categorical data. The association between variables was determined using χ^2^ and Fisher's exact tests. Statistical significance was set at *P* < 0.05.

## Results

Five patients are included in this series, ages 4 to 7 (median, 6), 60% male and 40% female. The median follow-up for all patients was 35 months (range, 32-40 months). No patients had other comorbid illnesses, family history of cancer, or known predisposing genetic conditions. Their diagnoses included intracranial germ cell tumor-nongerminoma germ cell tumor (n = 1), MB (n = 1), pineoblastoma (n = 1), CNS embryonal tumor (n = 1), and ependymoma (n = 1).

All 5 patients received some surgical intervention before presentation at our center. Two patients (40%) had gross total resection of the primary tumor, 2 (40%) had subtotal resection, and 2 (40%) ventriculoperitoneal shunt insertion (Table 3). CSF cytology was positive in 2 patients (40%) and not reported in 3 (60%). One patient (patient 5) declined second surgery at our center on account of financial constraints. The median time from surgery to RT was 96 days (36-117 days) overall.

**Table 3:** Patient, tumor, and treatment characteristics

	Case 1	Case 2	Case 3	Case 4	Case 5
Age	6 (76 mo)	7 (90 mo)	4 (59 mo)	6 (78 mo)	5 (70 mo)
Sex	M	M	M	F	F
Diagnosis	Pineoblastoma	Medulloblastoma	ICGCT (NGGCT): Yolk sac tumor	CNS embryonal tumor (NOS)	Ependymoma
Pathology	Pineoblastoma	Medulloblastoma WHO grade IV	Yolk sac tumor AFP positive	IHC - NSE, CD99, vimentin positive CD20, CD34, Tdt, desmin MYF-4, synaptophysin, LCA negative	Ependymoma WHO II
Surgery	Craniotomy + VP shunt insertion and subtotal resection/ biopsy via right occipital transtentorial approach	Craniotomy + GTR	Craniotomy + VP shunt insertion	Craniotomy + GTR	Craniotomy + subtotal resection; second-look surgery declined
CSF cytology	Cytology - acellular smear; tumor markers - AFP, bHCG: low	Not done	Cytology - positive; tumor markers: elevated AFP	Not done	Not done
Duration from surgery to RT (days)	96	36	117	100	44
Risk stratification	High	High	High	High	High
Chemotherapy	Neo-adjuvant: Alternating IE-CE	Concurrent weekly vincristine; adjuvant	Neo-adjuvant CE alternating with IE x 6 cycles	Neo-adjuvant: Alternating IE-CE	None
CSI dose	36.000 Gy	36.000 Gy	36.000 Gy	36.000 Gy	36.00 Gy
Boost dose	18.000 Gy	18.000 Gy	18.000 Gy	18.000 Gy	19.800 Gy
Boost volume	Primary tumor (54 Gy)	Posterior fossa (54 Gy)	Primary tumor (54 Gy)	Posterior fossa (54 Gy)	Residual tumor (55.8 Gy)
Treatment duration	6 wk	8 wk	7 wk	6 wk	6 wk
Treatment interruption	No	Yes	No	No	No

*Abbreviations:* AFP = alpha feto-protein; bHCG = beta - human chorionic gonadotropin; CD = cluster of differentiation; CE = carboplatin/etoposide; CNS = central nervous system; CSF = cerebrospinal fluid; CSI = craniospinal irradiation; GTR = gross total resection; ICGCT = intracranial germ cell tumor; IHC = immunohistochemistry; IE = ifosfamide/etoposide; ; LCA = leukocyte common antigen; MYF = myogenin; NGGCT = non-germinoma germ cell tumor; NOS = not otherwise specified; NSE = neuron-specific enolase; RT = radiation therapy; VCP = ventriculoperitoneal; VP = ventriculoperitoneal; WHO = World Health Organization.

All patients completed the intended course of radiation with a median duration of 6 weeks (range, 6-8 weeks). There were no extensive treatment interruptions and no treatment abandonment in this set of patients. Four patients in this series had chemotherapy. Three patients received neo-adjuvant chemotherapy with alternating cycles of carboplatin/etoposide and ifosfamide/etoposide. One patient had concurrent weekly vincristine during RT followed by post-RT adjuvant vincristine/cisplatin/cyclophosphamide chemotherapy.

**Figure 4:**
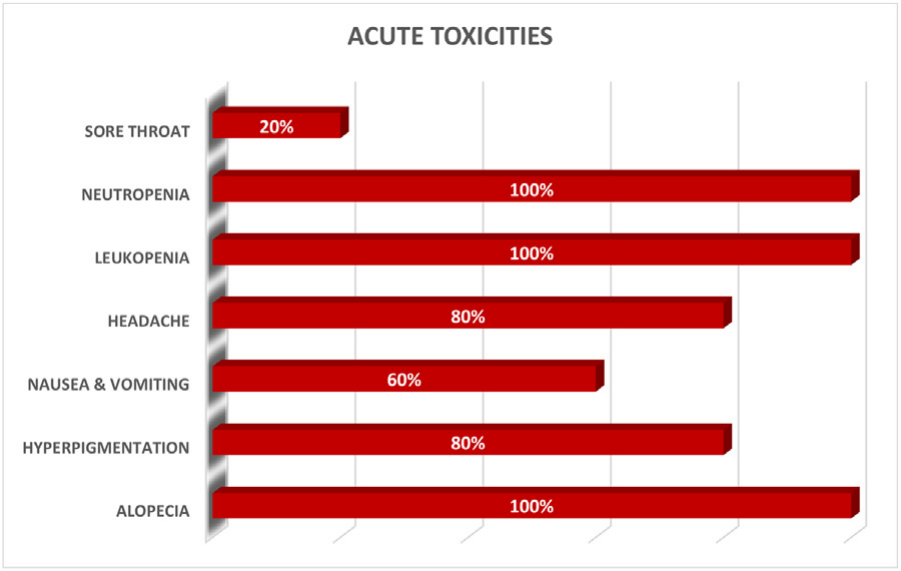
Acute toxicities. Acute side effects observed in the study participants.

Acute side effects included (Fig. 4): alopecia (100%), hyperpigmentation (80%), nausea and vomiting (60%), headache (80%), leukopenia (100%), anemia (100%), thrombocytopenia (40%), sore throat (20%), anorexia (60%), fatigue (80%), and weight loss (100%). All acute toxicity was graded as 1 or 2, and no severe toxicity was observed. No emotional distress or acute mental health disorders were recorded.

Four patients reported headache at least once from the start of treatment to 6 weeks post treatment. This was resolved with over-the-counter analgesics in all patients. Four patients were noted to have grade 1 hyperpigmentation in the skin of the scalp, back, or both. No medical intervention was necessary, and caregivers were counseled on skincare, such as to avoid scrubbing the skin or showering with scalding hot water, use of emollient body wash or lotion, and keeping the skin clean and dry. All 5 patients were noted to experience some hair loss over the scalp. They were counseled on the possibility of this before treatment and counseled on skin care as outlined previously. One patient reported sore throat ( CTCAE grade 2 esophagitis), which was resolved with over-the-counter analgesics.

Other acute side effects reported were nausea and vomiting in 3 patients (managed with hydration and ondansetron), anorexia and weight loss (managed with nutrition counseling by the radiation oncologist and PediaSure supplement recommendations), and fatigue.

With a median follow-up of 35 months, all patients had an acceptable resolution of acute toxicity. No patient had recurrence of symptoms, and all were alive at the last contact, excluding the patient lost to follow-up. Long-term toxicities and cancer outcomes are being monitored.

## Discussion

Here we report the first series of pediatric patients to receive VMAT-based CSI in a cancer center in Nigeria. Although CSI has long been employed in the management of primary CNS malignancy or spinal dissemination in Nigeria, VMAT is a novel technique that is not readily available in most of sub-Saharan Africa.^15^^,^^16^^,^^25^ This report demonstrates the feasibility of safely implementing advanced RT techniques for pediatric patients and serves as a model for the region.

The time from surgery to RT in this study ranged from 36 to 117 days, which is higher than the average 4 to 5 weeks considered ideal timing by clinicians.^26^^,^^27^ Although there is no standard time to postoperative RT, trials have shown that intervals longer than 22 weeks lead to poorer outcomes in patients.^27^ The increase in treatment lag time in this study can be attributed to the barriers in accessing RT unique to resource-limited settings. These barriers include geographic distance to the RT center, financial constraints, and lack of cohesiveness of the multidisciplinary care team, as different aspects of patients’ treatments are far flung and received at different centers and locations.^28^^,^^29^

With a median follow-up of 35 months, we report the presence of and resolution of acute toxicity, all grades 1 and 2. Although we saw a similar frequency of acute hematologic adverse events, our data are comparable to a similar study by Lopez et al.^30^ which showed low rates of severe acute toxicities with RT. Known predictors of severity of toxicity include age, higher doses of radiation, and neoadjuvant chemotherapy as predictors of hematologic toxicities observed during CSI.^31^^,^^32^ Other authors have documented that concurrent vincristine administration is not associated with hematologic toxicities.^31^

We also observed no severe nonhematologic toxicity in our patient cohort. Specifically, we observed mild gastrointestinal (GI) toxicities (≤grade 2), including sore throat, nausea, and vomiting. Further, weight loss was generally mild, grade 1 to 2, corroborating our observation of mild GI toxicity. Multiple studies correlate weight loss as a surrogate for evaluating GI toxicities in patients undergoing CSI.^30^^,^^32^^,^^33^ In contrast to other studies using helical tomotherapy, we observed a greater frequency of headache, alopecia, and hyperpigmentation. Schiopu et al^34^ observed alopecia and hyperpigmentation in 37.8% and 8.9% of patients, respectively. The seemingly significant difference in the percentages may be explained by the sample size difference: 5 in the present study versus 45 in Schiopu et al, although severity did not exceed grade 2.

The use of VMAT CSI in the management of pediatric solid tumors can be described as feasible in an LMIC based on this study. This mode of radiation treatment does not require a specialized machine. Thus, it can be provided to patients using the available LINACs, eliminating increased financial costs. Several studies have demonstrated the feasibility of treating average/standard risk patients with reduced dose CSI without having a negative effect on overall survival outcomes,^20^^,^^35^^,^^36^ especially with the addition of chemotherapy.^37^ In the absence of accurate risk stratification of patients, these cases can be treated as high risk.^37^ Limitations of pathologic testing in LMIC lead to upstaging of patients and potential overtreatment. In the series, all patients were staged as high risk because of lack of pathologic information that could potentially reduce dose required for treatment.

A possible barrier to the implementation of this treatment modality is the requirement of sedation for pediatric patients. Although none of the patients in this series were sedated, the lack of specialists in pediatric anaesthesiology, cost of treatment, and unavailability of anesthetic agents are factors that will hamper the use of VMAT CSI in resource-limited settings.^33^^,^^38^

RT for pediatric patients costs an average of $750 to $1500 in our center. At our center, treatment fees are paid in bulk before the start of treatment and based on diagnosis and treatment indication (radical or palliative) and not technique or number of fractions. As such there is no difference in cost to the patient for VMAT, IMRT, or three-dimensional conformal radiation therapy (3DCRT). This billing system was found to be more appropriate for our setting, where resources are limited and 90% of costs are paid out-of-pocket. This system was implemented to avoid treatment interruptions, as all fees have been paid upfront, and it frees oncologists to make decisions on dosing regimen and technique based purely on clinical indication without the pressure of worrying about patient finances.

In patients who need sedation, the cost climbs significantly by an average of $1000 for the duration of treatment. Although this may seem low in relation to costs of treatment in high-income countries, in the context of the local currency and exchange rate, these amounts are in the range of millions. When viewed against the background of the minimum wage and a general lack of health care insurance coverage, these costs are often paid fully out-of-pocket by families and can be financially distressing.^24^ The additional cost of sedation is further motivation to reduce the need for sedation for pediatric patients on RT in our setting.

As such, additional time is required to make the child comfortable for treatment without sedation. This is done by the radiation oncologist and therapy technologist. This time is not accounted for in resource allocation and is a significant investment by the pediatric radiation team. This barrier can potentially lead to increased treatment times, further translating to increased cost of treatments for patients in countries where the majority of health care expenditure is out-of-pocket.^33^ Hence, the avoidance of anesthesia reduces treatment costs and the overall time spent in the center by eliminating the time spent preparing to sedate the patient before the actual treatment and the time spent resuscitating and monitoring the patient afterward.

A limitation of this study is the small sample size and limited follow-up to date. Long-term follow-up for outcomes and side effects is ongoing. Another limitation is the lack of a centralized referral system in the country. All the patients in this case series had undergone some prior diagnostic, surgical, or medical intervention at different centers, some of them out-of-state, before referral to our center for RT. Information on previous interventions were retrieved from referral letters and medical reports presented upon their arrival.

The absence of critical pieces of pathologic information in this case series led to upstaging as high risk for all the patients. This is a common limitation in our setting, and patients who can have de-escalated dosing of RT may be overtreated.

Safe implementation of VMAT for CSI in low- and middle-income settings is resource intense but feasible. Although the expertise, equipment, and economic requirements are steep, investment in advanced RT treatment techniques will hopefully reduce side effects and improve outcomes.

## Conclusion

Our study demonstrates that LMIC nations like Nigeria can safely implement advanced techniques and technologies in the treatment of pediatric brain tumors. Additional follow-up will be needed to determine how survival outcomes and late toxicity data compare with reported outcomes.

## Disclosures

The authors declare that they have no known competing financial interests or personal relationships that could have appeared to influence the work reported in this paper.
